# SegAE: Unsupervised white matter lesion segmentation from brain MRIs using a CNN autoencoder

**DOI:** 10.1016/j.nicl.2019.102085

**Published:** 2019-11-09

**Authors:** Hans E. Atlason, Askell Love, Sigurdur Sigurdsson, Vilmundur Gudnason, Lotta M. Ellingsen

**Affiliations:** a Department of Electrical and Computer Engineering, University of Iceland, Reykjavik, Iceland; b Department of Medicine, University of Iceland, Reykjavik, Iceland; c Department of Radiology, Landspitali - University Hospital, Reykjavik, Iceland; d The Icelandic Heart Association, Kopavogur, Iceland; e Department of Electrical and Computer Engineering, The Johns Hopkins University, Baltimore, MD, USA

**Keywords:** CNN, Segmentation, White matter hyperintensity, Brain, Autoencoder, Deep learning

## Abstract

White matter hyperintensities (WMHs) of presumed vascular origin are frequently observed in magnetic resonance images (MRIs) of the elderly. Detection and quantification of WMHs is important to help doctors make diagnoses and evaluate prognosis of their elderly patients, and once quantified, these can act as biomarkers in clinical research studies. Manual delineation of WMHs can be both time-consuming and inconsistent, hence, automatic segmentation methods are often preferred. However, fully automatic methods can be challenging to construct due to the variability in lesion load, placement of lesions, and voxel intensities. Several state-of-the-art lesion segmentation methods based on supervised Convolutional Neural Networks (CNNs) have been proposed. These approaches require manually delineated lesions for training the parameters of the network. Here we present a novel approach for WMH segmentation using a CNN trained in an unsupervised manner, by reconstructing multiple MRI sequences as weighted sums of segmentations of WMHs and tissues present in the images. After training, our method can be used to segment new images that are not part of the training set to provide fast and robust segmentation of WMHs in a matter of seconds per subject. Comparisons with state-of-the-art WMH segmentation methods evaluated on ground truth manual labels from two distinct data sets and six different scanners indicate that the proposed method works well at generating accurate WMH segmentations without the need for manual delineations.

## Highlights

• A convolutional neural network designed for unsupervised segmentation of tissue and white matter hyperintensities (WMHs) in brain MRIs.
• Evaluations were conducted on two distinct datasets comprising data from six different scanners, all with ground truth manual WMH label masks.
• The method compares favorably to existing WHM segmentation methods and is fast and robust to highly variable WMH lesion load and atrophy in the brains of elderly subjects.

## 1 Introduction

White matter lesions that appear hyperintense in T2-weighted (T2-w) and Fluid Attenuated Inversion Recovery (FLAIR) images, and can appear hypointense in T1-weighted (T1-w) images, are frequently observed in magnetic resonance images (MRIs) of the elderly. They are often attributed to cerebral small vessel disease (Wardlaw, Smith, Biessels, Cordonnier, Fazekas, Frayne, Lindley, T O’Brien, Barkhof, Benavente, et al., 2013, Wardlaw, Valdés Hernández, Muñoz-Maniega, 2015) and termed white matter hyperintensities (WMHs) of presumed vascular origin (Wardlaw et al., 2013). WMHs of presumed vascular origin are generally associated with cognitive decline and dementia, such as Alzheimer’s disease and vascular dementia, or a mixture thereof (Debette, Markus, 2010, Wu, Sharrett, Gottesman, Power, Mosley, Jack, Knopman, Windham, Gross, Coresh, 2019). Detection and quantification of WMHs could have clinical usefulness in diagnostic workup of patients with mild dementia, and to evaluate the patient’s prognosis. Such measurements could also be used as a biomarker for neurodegenerative diseases in clinical research studies (Lee, Viqar, Zimmerman, Narkhede, Tosto, Benzinger, Marcus, Fagan, Goate, Fox, et al., 2016, Wardlaw, Valdés Hernández, Muñoz-Maniega, 2015). In any case, failure to account for WMHs in automatic segmentation methods can interfere with the segmentation of other brain structures, and thus, it is critical to be able to robustly identify these features (González-Villà et al., 2019).

The currently accepted gold standard in WMH segmentation is manual delineation by an expert in neuroanatomy. However, human raters can have great intra- and inter-rater variability (Carass et al., 2017) and acquiring such delineations is both time-consuming and expensive, making it impractical for analysis in large-scale studies. Automatic segmentation methods are necessary for such studies and they can be broadly classified into supervised and unsupervised methods. Supervised methods, most notably based on Convolutional Neural Networks (CNNs), have recently achieved state-of-the-art results on various datasets (Carass, Roy, Jog, Cuzzocreo, Magrath, Gherman, Button, Nguyen, Prados, Sudre, et al., 2017, Guerrero, Qin, Oktay, Bowles, Chen, Joules, Wolz, Valdés-Hernández, Dickie, Wardlaw, et al., 2018, Kamnitsas, Ledig, Newcombe, Simpson, Kane, Menon, Rueckert, Glocker, 2017, Kuijf, Biesbroek, de Bresser, Heinen, Andermatt, Bento, Berseth, Belyaev, Cardoso, Casamitjana, et al., 2019). A frequently used CNN architecture is the U-net (Ronneberger et al., 2015), which is a fully convolutional network with skip connections between the downsampling and upsampling paths. Supervised CNNs usually need a considerable amount of manually delineated lesion labels for training to capture the possible lesion variation in unseen images. This can be a major drawback in these methods, since new manually delineated segmentations are often needed to segment new data sets from different sites due to pulse sequence or scanner differences, and such delineations may be impractical to acquire. Attempts to reduce the number of manually delineated masks needed for training include transfer learning (Ghafoorian et al., 2017), and generative adversarial networks (GANs) (Bowles, Chen, Guerrero, Bentley, Gunn, Hammers, Dickie, Hernández, Wardlaw, Rueckert, Frid-Adar, Klang, Amitai, Goldberger, Greenspan, 2018).

Unsupervised methods typically involve modeling of MRI brain tissue intensities. These include methods that obtain WMH lesions as outliers of tissue segmentation (Lladó et al., 2012) and approaches that use specific features of lesions, such as voxel intensity and appearance  (Khayati, Vafadust, Towhidkhah, Nabavi, 2008, Lladó, Oliver, Cabezas, Freixenet, Vilanova, Quiles, Valls, Ramió-Torrentà, Rovira, 2012, Tomas-Fernandez, Warfield, 2015). Clustering or unmixing methods could potentially be used on a per image basis if a given image has enough WMH lesion load (Chai et al., 2010). One cluster may then correspond to WMHs in the brain. However, the number of WMH lesions and their location can vary greatly between subjects, and in the case of an image with no lesions, no cluster would correspond to the lesion class. Furthermore, modelling tissue intensities can be challenging because tissue intensities of MRIs are not always consistent within the image, e.g., due to inhomogeneity artifacts and partial volume effects. FLAIR images are the structural sequence from which WMHs are usually most easily distinguished (Wardlaw et al., 2015), however, various artifacts or poor skull-stripping can lead to high-intensity regions in FLAIR images (Krupa and Bekiesińska-Figatowska, 2015) that could potentially be incorrectly classified as WMHs. Another unsupervised approach that has been proposed in the literature is to detect WMH lesions as outliers of pseudo-healthy synthesized images (Baur, Wiestler, Albarqouni, Navab, 2019, Bowles, Qin, Guerrero, Gunn, Hammers, Dickie, Hernández, Wardlaw, Rueckert, 2017). A training data set with healthy brains (no lesions) is required to model normality in these approaches, such that lesions can be detected either as outliers or as results of large reconstruction errors (Baur, Wiestler, Albarqouni, Navab, 2019, Bowles, Qin, Guerrero, Gunn, Hammers, Dickie, Hernández, Wardlaw, Rueckert, 2017). This is usually not the case when analyzing brain MRIs of subjects older than 65 years old, where around 95% of the population will be expected to have WMHs (Longstreth et al., 1996).

A combination of linear unmixing and a neural network autoencoder has been proposed in hyperspectral unmixing of remote sensing images (Palsson et al., 2018). The purpose of these methods is to simultaneously find the amount of materials (such as water, grass, soil, etc.) in every pixel of the image and its contribution to the image intensity. By viewing various MRI sequences as “multispectral data” and individual brain tissues as different materials [such as WMHs, white matter (WM), gray matter (GM), and cerebrospinal fluid (CSF)], one can adopt such strategies into medical imaging. In our proposed segmentation method we model the intensities of multiple MRI sequences as weighted sums of the segmentations of materials present in the MRIs, as estimated by a convolutional autoencoder from the corresponding MRI sequences.

In hyperspectral unmixing, the number of image channels is usually much higher than the number of materials to be estimated, however, in the case of MRI, fewer MR sequences — or MRI modalities — are available to restrict this ill-posed inverse problem; hence, a regularization is needed. Our proposed CNN has a U-net like architecture, but with an additional linear layer and parameter constraints to perform linear unmixing. This allows the network to generalize the unmixing of materials from a set of training data. The network is trained using a scale-invariant cost function with regularization to determine the materials from which to reconstruct the MRIs. The training images are inhomogeneity corrected during the training phase, such that the CNN learns to segment new images in presence of inhomogeneity artifacts. After training the CNN autoencoder on a training set with a sufficient lesion load, it can be used to directly segment images that were not part of the training set. The segmentations are consistent for new images regardless of lesion load and location. We will hereafter refer to the proposed method as the Segmentation Auto-Encoder (SegAE).

A preliminary version of SegAE was recently published in conference format (Atlason et al., 2019). Here we present substantial improvements to this prior work by means of: (1) A scale-invariant loss function and a regularizer, (2) more MR sequences contributing to the calculation of the loss function, (3) an inhomogeneity correction performed during the training phase; and (4) a more extensive evaluation of the method on two data sets from 6 distinct scanners, all with ground truth manual lesion labels. Furthermore, a comparison with the preliminary version is presented as Supplementary materials.

## 2 Materials

Two data sets were used for the evaluation of SegAE; MRIs from the AGES-Reykjavik study (Forsberg et al., 2017), and the WMH challenge (Kuijf et al., 2019) initiated at the International Conference on Medical Image Computing and Computer Assisted Intervention (MICCAI) 2017. We note that the MRIs in the WMH challenge originate from 5 different scanners.

### 2.1 The AGES-Reykjavik data set

The Age, Gene/Environment Susceptibility-Reykjavik Study (AGES-Reykjavik) was initiated in 2002 and was designed to examine risk factors, including genetic susceptibility and gene/environment interaction, in relation to disease and disability in old age (Harris et al., 2007). The AGES-Reykjavik Study cohort comprises 5764 participants (female and male, age 66–93 at first visit), 4811 of which underwent brain MRI (Forsberg et al., 2017). The MRIs were acquired using a dedicated General Electrics 1.5-Tesla Signa Twinspeed EXCITE system with a multi-channel phased array head cap coil. T1-w three dimensional (3D) spoiled gradient echo sequence (time to echo (TE): 8 ms, time repetition (TR): 21 ms, flip angle (FA): 30^∘^, field of view (FOV): 240 mm; 256 ×  256 matrix) with 0.94  ×  0.94  ×  1.5 mm^3^ voxel size and 110 slices; Proton Density (PD)/T2-w fast spin echo sequence (TE1: 22 ms, TE2: 90 ms, TR: 3220 ms, echo train length: 8, FA: 90^∘^, FOV: 220 mm^2^; 256 ×  256 matrix); and FLAIR sequence (TE: 100 ms, TR: 8000ms, time from inversion (TI): 2000 ms, FA: 90^∘^, FOV: 220 mm; 256 ×  256 matrix) with 0.86  ×  0.86  ×  3.0 mm^3^ voxel size and 54 slices.

For developmental purposes, we randomly selected 60 subjects from the cohort; 30 subjects for training, 5 for validation of model parameters, and 25 for testing. The developmental set consists of images from a second visit acquired 5 years later than the first visit on average. The WMHs in the test images were manually annotated by an experienced neuroradiologist to be used as ground truth data. The images used for validation were used to determine model architecture and hyperparameters based on visual inspection.

### 2.2 The WMH challenge data

We submitted our method to the WMH challenge (Kuijf et al., 2019), initiated at MICCAI 2017. This challenge aims to provide a benchmark for automatic segmentation of WMHs of presumed vascular origin and remains open and ongoing^1^. The publicly available training set includes 60 cases from 3 different scanners, while the challenge organizers keep 110 cases from 5 different scanners hidden for evaluation. The WMH challenge only provides T1-w and FLAIR sequences. Table 1 shows an overview of how the data set is separated into training and test sets. Table 2 shows scanning parameters for the 5 scanners.

**Table 1:** Overview of the WMH challenge data set, showing how the 170 cases from 5 scanners are separated into training (Tr.) and test (Te.) sets.

Institute	Scanner	Tr.	Te.
UMC Utrecht	3 T Philips Achieva	20	30
NUHS Singapore	3 T Siemens TrioTim	20	30
VU Amsterdam	3 T GE Signa HDxt	20	30
	1.5 T GE Signa HDxt	0	10
	3 T Philips Ingenuity (PET/MR)	0	10

**Table 2:** Scanning parameters for the WMH challenge data set, comprising data from 3 sites and 5 different scanners.

Scanner	Sequence	TR[ms]	TE[ms]	TI[ms]	Voxel size[mm^3^]	slices
Utrecht	3D T1-w	7.9	4.5	-	1.00 × 1.00 × 1.00	192
	2D FLAIR	11,000	125	2800	0.96 × 0.95 × 3.00	48
Singapore	3D T1-w	2300	1,9	900	1.00 × 1.00 × 1.00	N/A
	2D FLAIR	9000	82	2500	1.00 × 1.00 × 3.00	N/A
AMS GE3T	3D T1-w	7.8	3.0	-	0.94 × 0.94 × 1.00	176
	3D FLAIR	8000	126	2340	0.98 × 0.98 × 1.20	132
AMS GE1.5T	3D T1-w	12.3	5.2	-	0.98 × 0.98 × 1.50	172
	3D FLAIR	6500	117	1987	1.21 × 1.21 × 1.30	128
AMS PETMR	3D T1-w	9.9	4.6	-	0.87 × 0.87 × 1.00	180
	3D FLAIR	4800	279	1650	1.04 × 1.04 × 0.56	321

### 2.3 Preprocessing

**AGES-Reykjavik**: Images were preprocessed using standard preprocessing procedures: Resampling to 0.8  ×  0.8  ×  0.8 mm^3^ voxel size, rigid registration to the MNI-ICBM152 template (Fonov et al., 2009), and skull removal using MONSTR (Roy et al., 2017). For improved inhomogeneity correction in presence of WMHs and enlarged ventricles, the inhomogeneity correction was integrated into the method, as discussed in detail in Sections 3.3 and 3.4.

**WMH challenge**: Resampling of the WMH challenge data to 3 mm in the transversal direction and alignment of the 3D T1-w images to the FLAIR images was performed by the challenge organizers as described in Kuijf et al. (2019). Since the resolution of the training data and the manually delineated test data needs to be the same, we did not alter the resolution of any of the WMH challenge data. We performed skull removal of the training data set with MONSTR, however, for skull removal of unseen images in the testing phase (performed by the WMH challenge team), we developed a skullstripping U-net that was trained on the MONSTR brainmasks derived from the training set (see Supplementary materials). As for the AGES-Reykjavik data set, inhomogeneity correction was integrated into the segmentation method (see Section 3.3).

## 3 Methods

### 3.1 CNN architecture

The proposed method, SegAE, is an autoencoder with fully convolutional layers on three resolution scales. The input into SegAE consists of large three-dimensional (3D) patches of MRI sequences, such as FLAIR, T1-w, and T2-w images (see Section 3.5 for details on the training procedure). The autoencoder is constrained to reconstruct the corresponding image patches with a linear unmixing model,

(1) $${\hat{Y}}_{c}=\sum_{i=1}^{M}w_{i,c}S_{i},$$

where ${\hat{Y}}_{c}$ is one channel of the output, $w_{i,c}\in R_{\geq 0}$ are the weights, $S_{i}$ is the soft segmentation of materials (such as WMHs, WM, GM, CSF and meninges), M is the number of materials to be estimated, $S_{i}\geq 0$ and $\sum_{i=1}^{M}S_{i}=B,$ where B is a binary brainmask (1 for voxels on the brain, 0 for voxels outside the brain).

The non-negativity constraint and the sum-to-one constraint of S are enforced with a Softmax activation function. A patch-wise brainmask obtained by binarizing the input patches is applied after the Softmax function. The weighted sum is implemented with a 1x1x1 convolutional layer that is constrained to have non-negative weights and zero bias. With appropriate regularization (see Section 3.2), the Softmax-layer outputs a soft segmentation of the materials present in the images.

**Fig. 1:**
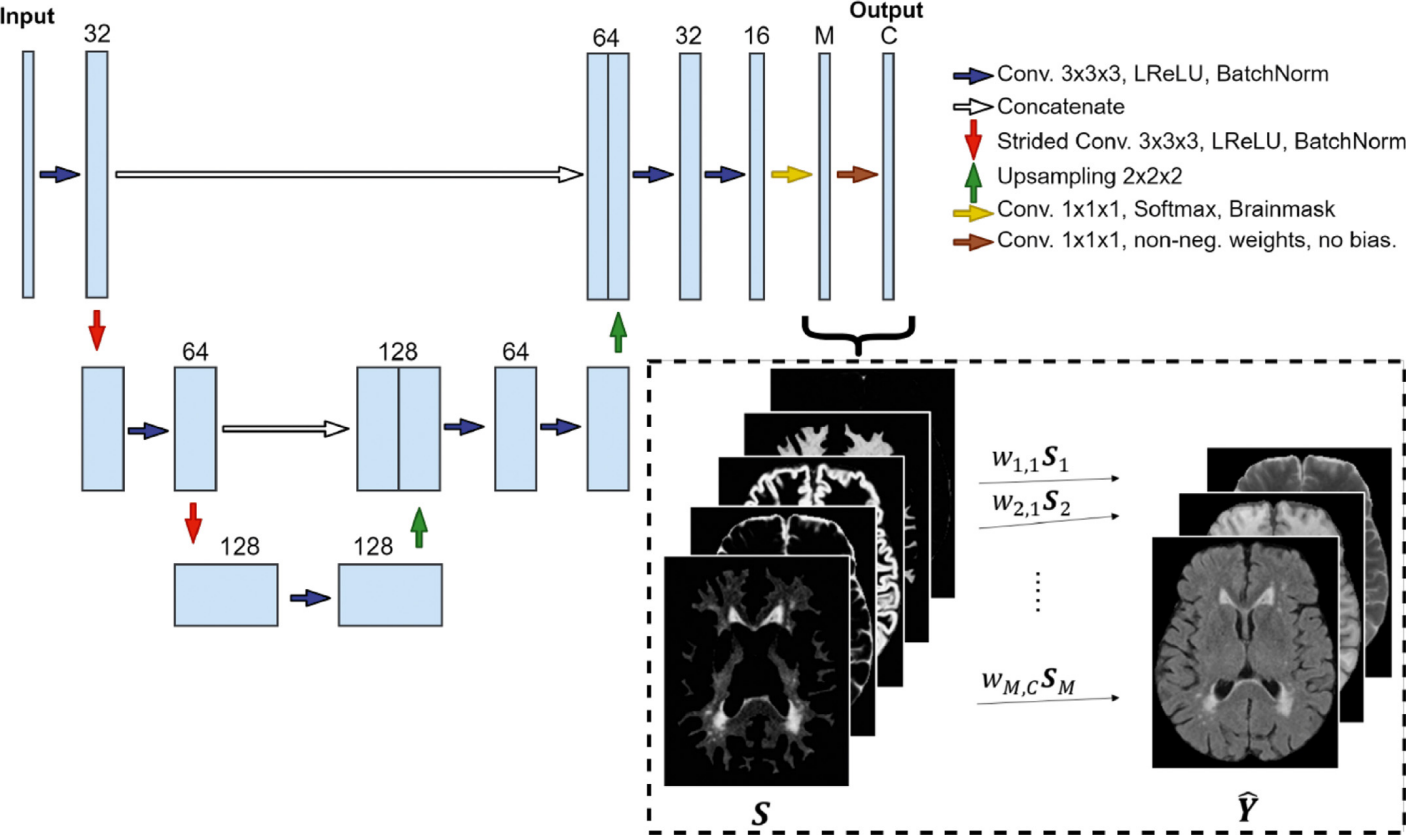
The proposed convolutional autoencoder architecture. The input comprises large 3D patches from different MRI sequences (FLAIR, T1-w, and T2-w are shown here). The final convolutional layer is restricted to have non-negative weights and zero bias for the reconstruction of the output patches $\hat{Y}$ to be a weighted sum of the Softmax outputs S. The number of output channels (one for each MRI sequence used) is denoted with *C* (C=3 in this case), and the number of materials to be estimated from the images is denoted with *M* (M=5 in this case).

The autoencoder consists of 3D convolutional layers followed by leaky rectified linear units (LReLU) activation functions and batch normalization layers. Downsampling is performed with 2 × 2 × 2 strided convolutions, and 2 × 2 × 2 upsampling is performed to obtain an output of the same size as the input. Skip connections are added between activations of the same spatial resolution from the downsampling to the upsampling paths. The CNN architecture is demonstrated in Fig. 1.

### 3.2 Loss and regularization

The Cosine proximity function,

(2) $$f\left(y,\hat{y}\right)=\frac{y\cdot \hat{y}}{{∥y∥}_{2}{∥\hat{y}∥}_{2}},$$

is used to construct a scale invariant loss function between the true patches Y and the predicted patches $\hat{Y}$:

(3) $$L\left(Y,\hat{Y}\right)=-\frac{1}{C}\sum_{c=1}^{C}\left(f\left(\mathrm{vec}\left(Y_{c}\right),\mathrm{vec}\left({\hat{Y}}_{c}\right)\right)+f\left(\mathrm{vec}\left(K*Y_{c}\right),\mathrm{vec}\left(K*{\hat{Y}}_{c}\right)\right)\right),$$

where *C* is the number of channels in Y and $\hat{Y},$ *K* is the 3D discrete Laplace operator

$$K_{1,3}=\left[\begin{array}{ccc} 0 & 0 & 0\\ 0 & 1 & 0\\ 0 & 0 & 0 \end{array}\right],K_{2}=\left[\begin{array}{ccc} 0 & 1 & 0\\ 1 & -6 & 1\\ 0 & 1 & 0 \end{array}\right],$$

and * denotes a convolution. Using the differential operator *K* in the loss function was found to improve robustness to the slowly varying tissue inhomogeneity.

Reconstructing the MRI sequences as weighted sums of the materials present in the images is an ill-posed inverse problem, since we have fewer MRI sequences than materials of interest, and hence, a regularization is needed. For this we add an activity regularization term to the loss function that penalizes the sum of Cosine proximity between the Softmax outputs,

(4) $$\Omega \left(S\right)=\frac{\alpha }{M}\sum_{i=1}^{M}\sum_{j=1}^{M}f\left(\mathrm{vec}\left(S_{i}\right),\mathrm{vec}\left(S_{j}\right)\right),$$

where *α* is the regularization parameter.

### 3.3 Inhomogeneity correction

**Fig. 2:**
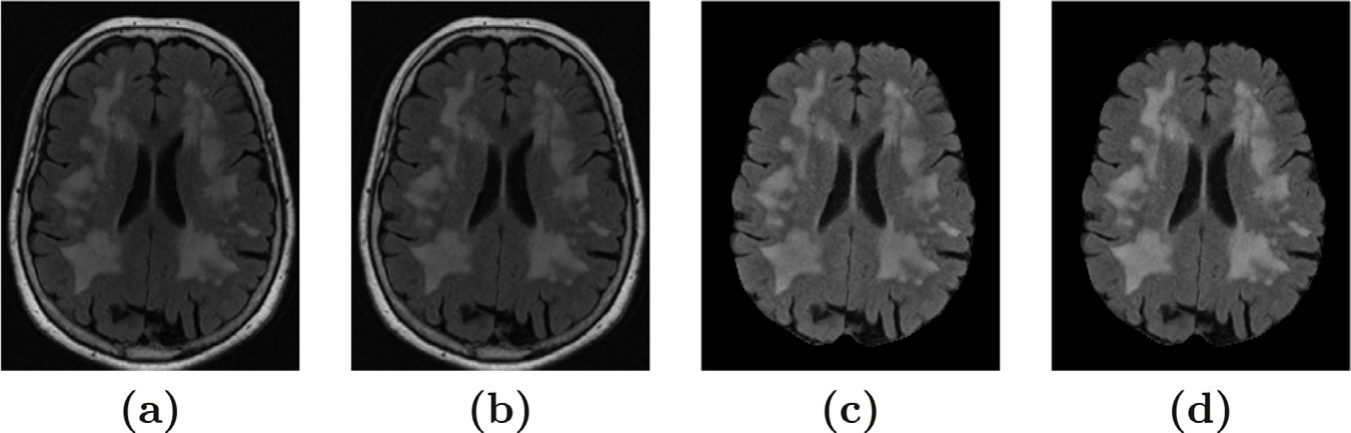
The figure shows the effect of N4 bias correction on a FLAIR image with a large lesion load. **(a)** The original FLAIR image before skullstripping; **(b)** after N4 bias correction (with skull); **(c)** After N4 bias correction (without skull); and **(d)** After skull-stripping and bias correction using pure-tissue probability mask.

A disturbance of the field homogeneity in MR scanners leads to low frequency signal artifacts in MRIs, which can make intensities of the brain tissues and WMHs overlap substantially. A widely used state-of-the-art method for inhomogeneity correction is the N4 bias correction method (Tustison et al., 2010). We observed that when N4 was directly applied to the FLAIR images (using 125 mm spline distance), it caused a substantial degradation of the lesion contrast in FLAIR images with a large lesion load (see Fig. 2 **(c)** and a more detailed comparison in Supplementary materials). Hence, to avoid this degradation, we alternated between using N4 bias correction and tissue segmentation to obtain “pure-tissue” probability masks, as suggested in Tustison et al. (2014). This improved the N4 bias correction, which in turn improved the next iteration of tissue segmentation. This iterative inhomogeneity correction was performed as follows:

We used SegAE to obtain a soft segmentation of tissues and WMHs, and created a pure-tissue probability mask using Softmax outputs that correspond to CSF, GM, and WM (excluding WMHs and meninges). Then we applied N4 bias correction using the pure-tissue probability mask so regions containing WMHs and partial volume effects would have minimal contribution to the inhomogeneity correction itself, leading to improved contrast between the WMH lesions and surrounding tissue. After the bias correction, SegAE was trained again using the original images as input, but now the bias corrected images were used for evaluation of the cost function during training. This way, SegAE learned to segment the original images without the need for intermediate inhomogeneity correction when evaluating new images, which were not in the training set.

### 3.4 Image enhancement

**Fig. 3:**
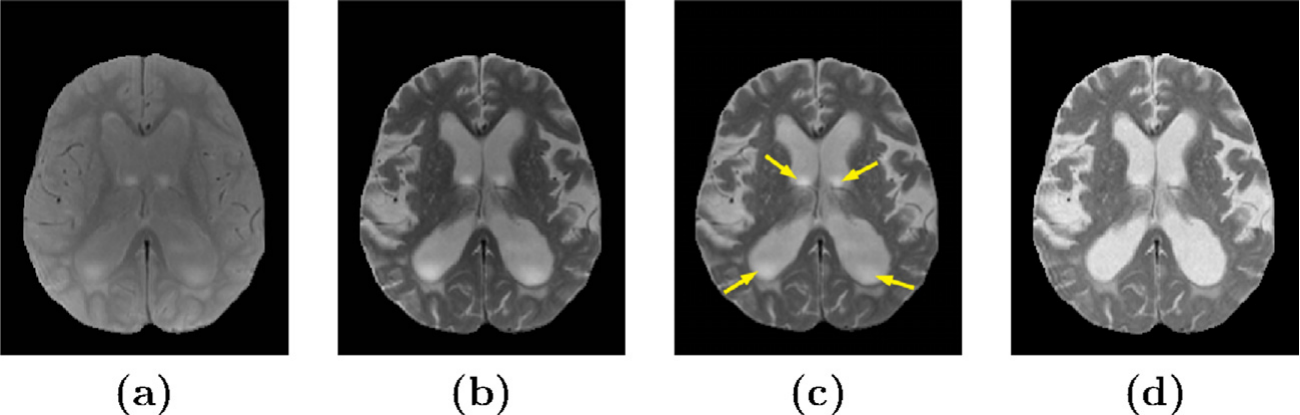
Image enhancement of a T2-w image using a PD image. **(a)** and **(b)** show the original PD and T2-w images, respectively; **(c)** shows the T2-w image after N4 bias correction with a pure-tissue probability mask; and **(d)** shows an enhanced image (T2_*PD*_).

**Fig. 4:**
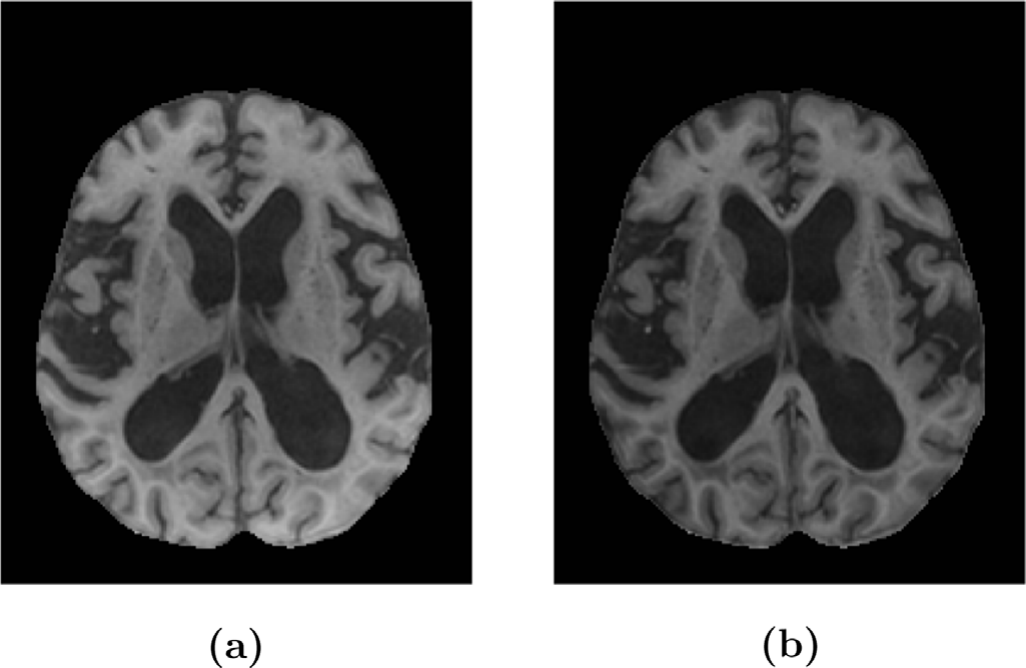
Image enhancement of a T1-w image using a PD image. **(a)** shows the original T1-w image and **(b)** shows an enhanced image (T1_*PD*_).

Presumed inhomogeneity artifacts within the CSF in T2-w and PD-w images were substantial in subjects with enlarged ventricles in the AGES-Reykjavik data set [see Fig. 3 **(a)** and **(b)**]. N4 bias correction using a pure-tissue probability mask was not sufficient to eliminate these artifacts (see Fig. 3 **(c)**, yellow arrows). We observed that inhomogeneity artifacts in the T2-w images and the PD-w images that were acquired simultaneously for each subject were highly correlated and the PD-w images had much lower contrast between the signals of interest. We synthesised enhanced images by multiplying the T1-w and T2-w images with the corresponding intensity transformed PD-w images (see Fig. 3 **(d)** and Fig. 4),

(5) $$I_{new}=I_{orig}⊙\left(\mathrm{Max}\left(I_{PD}\right)J-I_{PD}\right),$$

where $I_{new}$ is the enhanced image, $I_{orig}$ is the original T1-w or T2-w image, $I_{PD}$ is the original PD-w image, J is a matrix of ones of the same size as the PD-w image, and ⊙ denotes an element-wise multiplication. Multiplying the intensity transformed PD-w image with a T2-w image results in an image with a slightly degraded contrast of GM and WM compared to the original T2-w image, however, a contrast enhanced image can be acquired by multiplying it with the T1-w image (see Fig. 4). We will refer to the enhanced T1-w and T2-w images using PD-w images as T1_*PD*_ and T2_*PD*_, respectively.

### 3.5 Training

Two SegAE networks were constructed; one for the AGES-Reykjavik data set and one for the WMH challenge data set, since the AGES-Reykjavik data set comprises T1-w, T2-w, PD-w, and FLAIR images, while the WMH challenge data set only contains T1-w and FLAIR images. Table 3 gives an overview of the training data for each network. The number of Softmax output volumes in both models was 5, one for each material (WMH, WM, GM, CSF, and meninges). The regularization coefficient *α* was 0.0075 for the AGES-Reykjavik model and 0.02 for the WMH challenge model. Input images were intensity normalized by dividing by the 99th percentile of the non-zero elements of the image. The training images were cropped to the smallest cuboid containing the brain and patches from the images were acquired with a stride of 40 voxels. Only 50% of the extracted patches, which had the fewest background voxels, were used for training.

**Table 3:** Overview of the data used to train the two SegAE models for the AGES-Reykjavik (AGES-R.) and WMH challenge (WMH chall.) data sets.

SegAE model	Patch size	Modalities	Reconstruction
AGES-R.	80x80x80x3	T1, T2, FLAIR	T1_*PD*_, T2_*PD*_, FLAIR_*N*4_
WMH chall.	80x80x40x2	T1, FLAIR	T1_*N*4_, FLAIR_*N*4_

A GTX1080 Ti GPU was used to train the network for 80 epochs with a learning rate of 0.001 using the Adam optimizer (Kingma and Ba, 2014), with Nesterov momentum (Dozat, 2016), with $\beta_{1}=0.9,$ $\beta_{2}=0.999,$ schedule decay of 0.004, and a batch size of one. During training, Gaussian noise with a standard deviation of 0.05 and zero mean was added to the input patches, and different scalar values drawn from a Gaussian distribution with a mean value of 1 and standard deviation of 0.5 were multiplied with each channel of the input patches to improve the invariance of the network to possibly inconsistent normalization of unseen images. All weights of the convolutional network were initialized using Glorot uniform initialization (Glorot and Bengio, 2010) and biases were initialized as zero. LReLU activation functions had a slope of 0.1 for the negative part. Hyperparameters where chosen by trial and error. The regularization coefficient alpha was the main hyperparameter that needed to be estimated. The 5 validation images were visually inspected and alpha was determined based on the mixture between the estimated materials. Alpha was increased if there was too much mixture between segmentations and decreased if the segmentations were too coarse. The hyperparameters of the optimizer were set to default Tensorflow (Abadi et al., 2015) values.

### 3.6 Prediction and post-processing

**Fig. 5:**
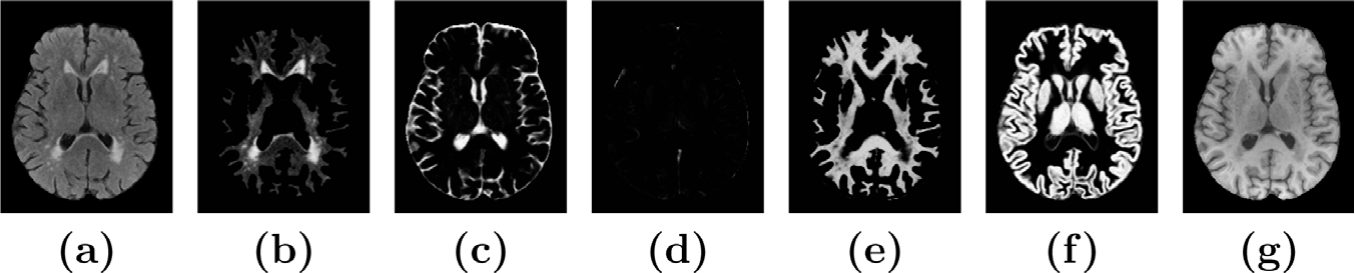
The tissue and WMH segmentation output from SegAE. **(a)** and **(g)** show the original FLAIR and T1-w images respectively, and **(b)-(f)** show the segmentations of WMH, CSF, meninges that remain after skullstripping, WM, and GM, respectively.

After training, the 5 Softmax output volumes (S in Fig. 1) were used for prediction, while the reconstructed images ($\hat{Y}$ in Fig. 1) were discarded. Prediction was performed with a stride of 40, and patches were assembled using the average of overlapping voxels. The assembled Softmax outputs from SegAE of a subject from the AGES-Reykjavik validation set revealed the segmentation of WMHs, GM, WM, CSF, and the meninges that remain in the image after skullstripping (see Fig. 5).

In this article we focus on automated segmentation of WMH lesions, and hence, only the output volume corresponding to the WMH segmentation is used in our evaluation of the method. The WMH segmentation for the AGES-Reykjavik model was binarized with a threshold of 0.5 and the WMH segmentation from the WMH challenge model was binarized with a threshold of 0.87, as determined with Bayesian optimization for maximizing the average Dice Similarity Coefficient (DSC) (Dice, 1945) on the WM challenge training data (Bergstra et al., 2013), and structures smaller than 3 voxels were removed from the segmentation results from the WMH challenge data due to noise in the cerebellum.

### 3.7 Evaluation metrics

For each test subject the following similarity metrics were computed to quantify the performance of SegAE and the competing methods compared to manually delineated lesions in the test cases:

• *Absolute Volume Difference (AVD)*
  The absolute difference in volumes divided by the true volume. Defined as $\frac{|V_{T}-V_{P}|}{V_{T}},$ where *V_T_* and *V_P_* denote the volumes of the manually delineated masks and predicted masks, respectively. Lower AVD indicates a more accurate prediction of WMH lesion volume.
• *Dice Similarity Coefficient (DSC)* (Dice, 1945)
  A measure of overlap between the ground truth and predicted segmentations. Using the true positives (TP), false positives (FP), and false negatives (FN) from the confusion matrix, DSC is defined as $\frac{2TP}{2TP+FP+FN},$ and takes values in the range [0, 1]. A DSC of 1 indicates a perfect overlap.
• *Modified Hausdorff distance (H95)*
  Hausdorff distance measures the longest distance one has to travel from a point in one set to a point in the other set, defined as:
  $$d_{H}\left(X,Y\right)=\max\left\{\;\underset{x\in X}{\sup}\underset{y\in Y}{\inf}d\left(x,y\right),\;\underset{y\in Y}{\sup}\underset{x\in X}{\inf}d\left(x,y\right)\;\right\}\text{,}\;$$
  where *d*(*x, y*) denotes the distance between *x* and *y*, sup denotes the supremum and inf the infimum. Here the 95th percentile is used instead of the maximum distance, since the Hausdorff distance is sensitive to outliers. Lower H95 scores indicate better performance.
• *Lesion-wise true positive rate (L-TPR)*
  Let *N_T_* be the number of individual WMH lesions in the ground truth mask (*T*), and *N_P_* be the number of correctly detected lesions after comparing the overlap of the predicted mask (*P*) to *T*. An individual lesion is defined as a 3D connected component. Then the lesion-wise true positive rate (L-TPR) is defined as $\frac{N_{P}}{N_{T}}$. Higher L-TPR indicates better performance.
• *Lesion-wise F1-score (L-F1)*
  Let *N_P_* be the number of correctly detected lesions after comparing *P* to *T. N_F_* is the number of incorrectly detected lesions in *P*. An individual lesion is defined as a 3D connected component, and L-F1 is defined as $\frac{N_{P}}{N_{P}+N_{F}}$. Higher L-F1 indicates better performance.

Finally, for the AGES-Reykjavik test set, a best linear fit was identified between the predicted and manually delineated volumes and the Pearson’s correlation coefficient (*r*) was used for comparison.

### 3.8 Comparison segmentations for the AGES-Reykjavik data set

The WMHs in a total of 25 subjects were manually delineated by a neuroradiologist to be used as ground truth lesion segmentations for evaluation of the proposed method. We compared the proposed method with three state-of-the-art methods; two publicly available WMH segmentation methods, i.e., the Lesion Growth Algorithm (LGA) (Schmidt et al., 2012) and the Lesion Prediction Algorithm (LPA) (Schmidt, 2017) as implemented in the LST toolbox^2^ version 2.0.15, and one method developed previously for the AGES-Reykjavik data set based on an artificial neural network classifier (ANNC) (Sigurdsson et al., 2012):

• *LGA* segments WMHs from T1-w and FLAIR images. A CSF, GM and WM segmentation is first obtained from the T1-w image and combined with FLAIR image intensities for calculation of WMH belief maps. The belief maps are thresholded by a pre-chosen threshold (*κ*) for an initial binary map, which is grown to include voxels that appear hyperintense in the FLAIR image for a final lesion probability map (Schmidt et al., 2012). We used κ=0.1 as determined by the result on our 5 validation images.
• *LPA* segments WMHs from a FLAIR image. LPA includes a logistic regression model trained on MRIs of 53 MS patients with severe lesion patterns obtained at the Department of Neurology, Technische Universität München, Munich, Germany. As covariates for this model a similar lesion belief map as for LGA was used as well as a spatial covariate that takes into account voxel specific changes in lesion probability. This model provides an estimated lesion probability map that can be thresholded for a WMH segmentation (Schmidt, 2017).
• *ANNC* is an artificial neural network classifier in the four dimensional intensity space defined by the four sequences (FLAIR, T1-w, PD-w, and T2-w) that was previously developed to obtain WMHs, GM, WM, and CSF segmentation for the AGES-Reykjavik MRIs. The input is the voxelwise intensities of FLAIR, T1-w, T2-w, and PD-w images and the classifier was trained on 11 manually annotated subjects (Sigurdsson et al., 2012).

## 4 Results

### 4.1 Evaluation on the AGES-Reykjavik data set

**Fig. 6:**
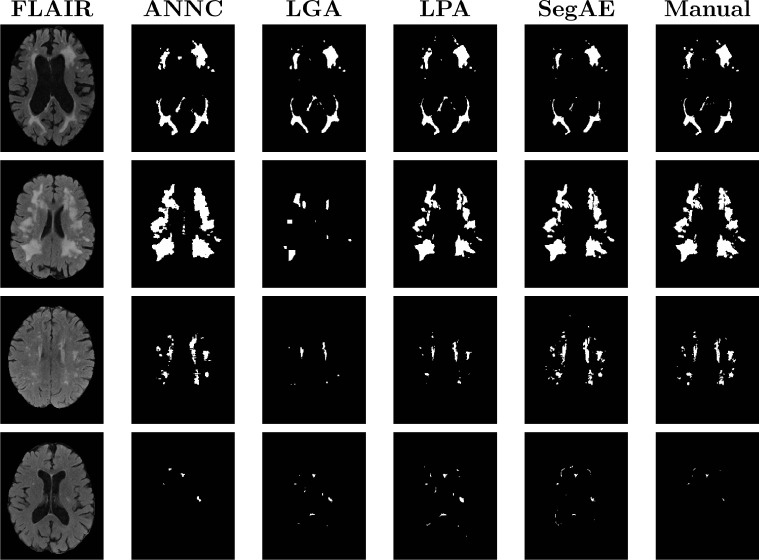
Visual comparison of the four methods with a manual rater for four different subjects, two with the largest and second largest lesion load (1st and 2nd row), one with a medium lesion load (3rd row), and one with the smallest lesion load (4th row).

Fig. 6 visually demonstrates the performance of the methods on four test images; two with the largest and second largest lesion load (1st and 2nd row), one with a medium lesion load (3rd row), and one with the smallest lesion load (4th row).

**Fig. 7:**
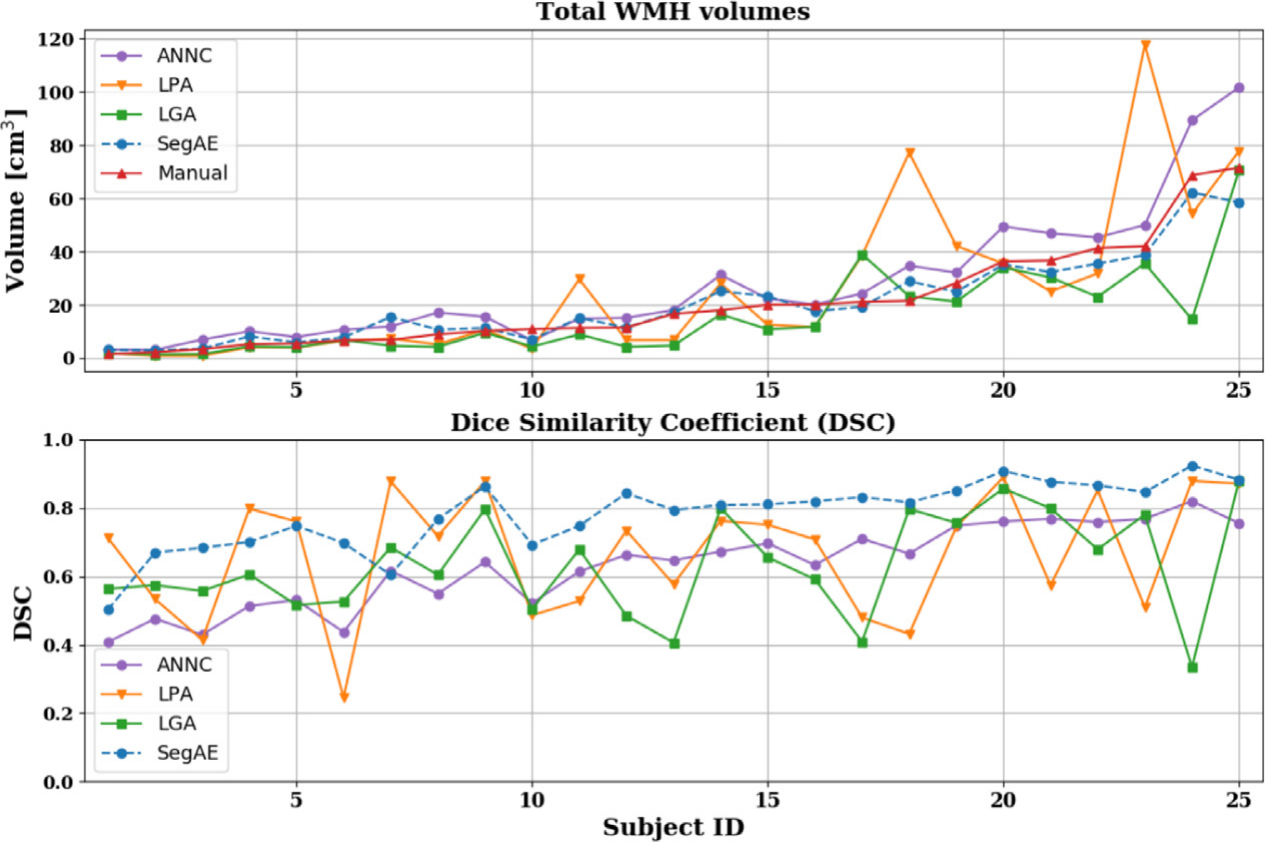
The top graph shows the overall WMH volume for the manual masks (red) and masks generated by ANNC (purple), LPA (orange), LGA (green), SegAE (blue, dotted), ordered by the volume of the manual masks. The bottom graph shows the DSC for the same methods compared with the manual masks.

Table 4 shows the mean and standard deviation of the DSC, H95, AVD, L-TPR, and L-F1 for each of the four methods. We used a paired Wilcoxon signed-rank test to obtain the p-values for determining statistical significance. We computed the total WMH volume estimated by the four methods and compared with the volume of the manual masks (see Fig. 7, top), as well as corresponding DSC of the four methods against the manual masks (see Fig. 7, bottom). The total WMH volume and DSC for every test subject is ordered by the volume of the manual masks (small lesion load on the left and large lesion load on the right side of the figure) for a direct comparison of DSC for different WMH lesion loads.

**Table 4:** AGES-Reykjavik results. The mean and standard deviation for each of the evaluation metrics. Asterisk (*) denotes values that are significantly different from SegAE (*p* < .01), and bold figures denote the best result for each metric.

Method	DSC	H95	AVD	L-TPR	L-F1
ANNC	0.62 ( ± 0.13)*	10.16 ( ± 10.40)	60.49 ( ± 29.75)*	0.44 ( ± 0.12)*	0.39 ( ± 0.10)
LGA	0.66 ( ± 0.15)*	15.22 ( ± 9.93)	**26.50** ( ± 23.58)	0.29 ( ± 0.12)	0.36* ( ± 0.11)
LPA	0.66 ( ± 0.19)	**9.20** ( ± 6.56)	62.28 ( ± 73.75)	0.53 ( ± 0.27)	0.40 ( ± 0.20)
SegAE	**0.77** ( ± 0.11)	10.97 ( ± 11.45)	33.31 ( ± 36.30)	**0.64** ( ± 0.19)	**0.47** ( ± 0.09)

**Fig. 8:**
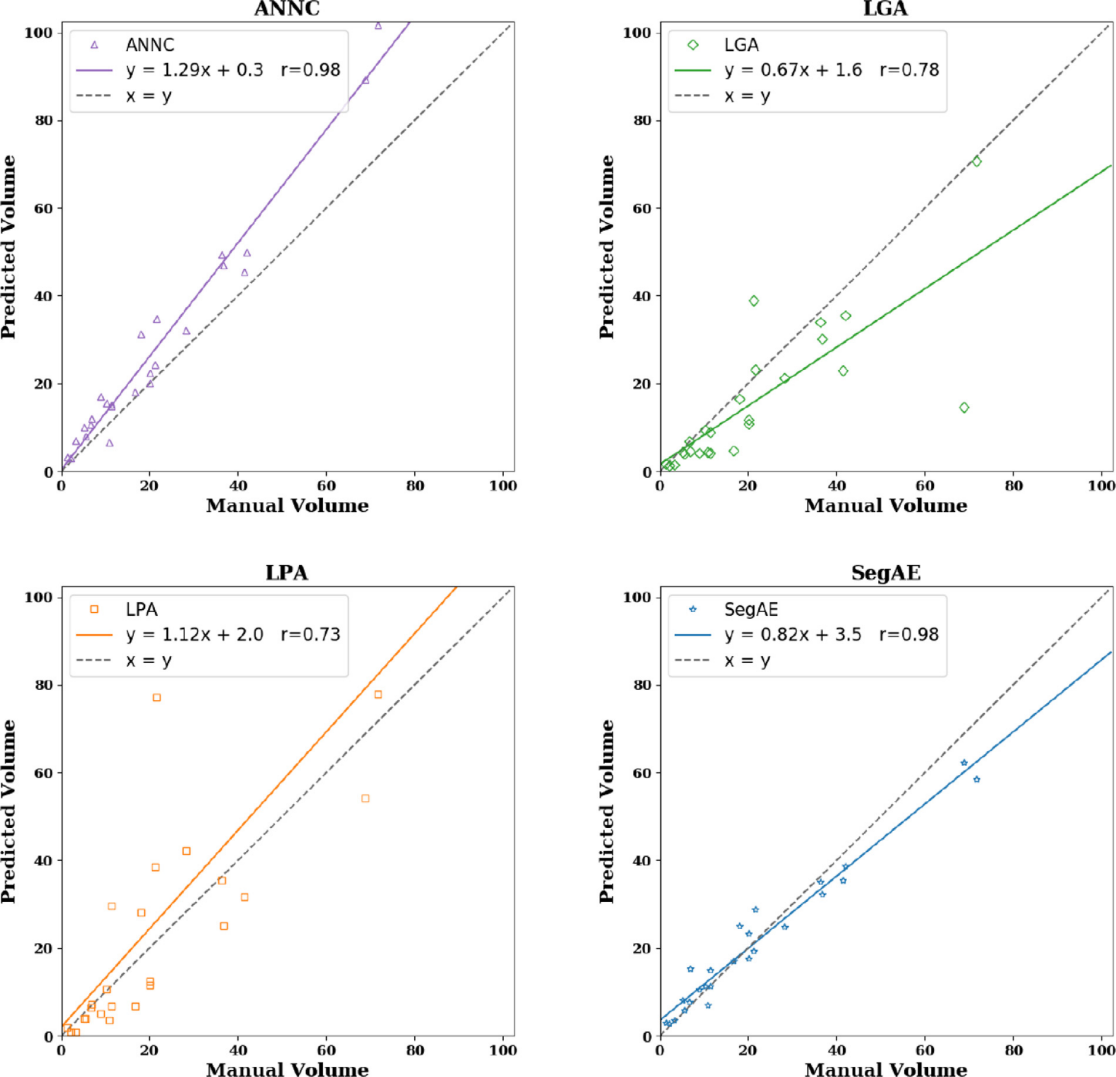
Predicted lesion volumes versus manual lesion volumes for the four methods. The solid lines show a linear fit of the points and the dashed black line has unit slope. Numbers are in cm^3^. Slope, intercept, and Pearson’s correlation coefficient between manual and predicted masks can be seen for the different methods.

Scatter plots showing predicted lesion volumes versus manual lesion volumes for the four methods, as well as the best linear fit and correlation coefficient, can be seen in Fig. 8. ANNC and SegAE achieve r=0.98, while LGA and LPA have r=0.78 and r=0.73, respectively.

### 4.2 Evaluation on the WMH challenge data set

**Fig. 9:**
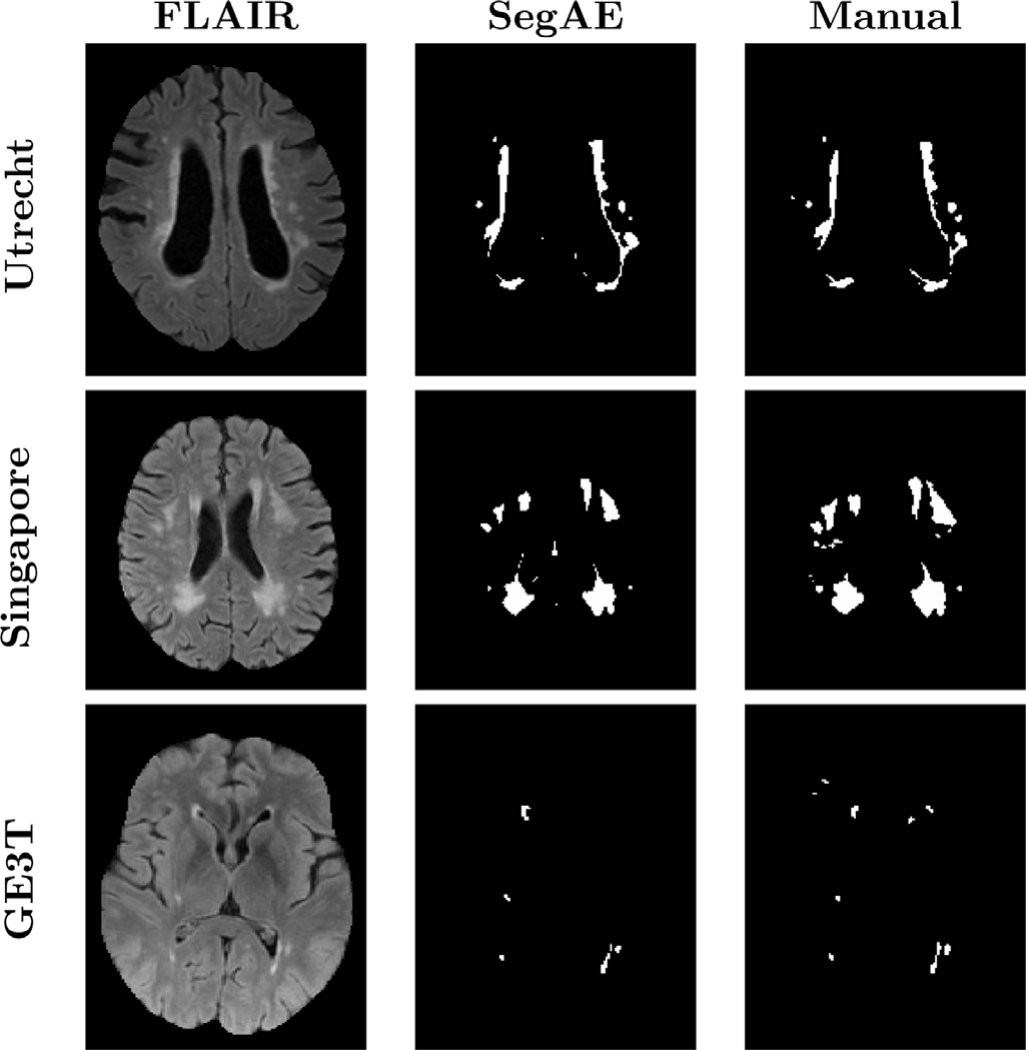
Visual comparison between the WMH segmentation of SegAE and the manually delineated masks for subjects in the WMH challenge training set. The top row shows the first subject (ID: 0) from the Utrecht scanner, the middle row shows the first subject from the Singapore scanner (ID: 50) and the bottom row shows the first subject in the GE3T scanner (ID: 100).

Fig. 9 shows a visual comparison between the WMH segmentation of SegAE and the manually delineated masks for 3 subjects in the WMH challenge training set. Table 5 shows the average AVD, DSC, Hausdorff distance, L-TPR, and L-F1 of SegAE on one test data from each of the five scanners, and a weighted average of the scores achieved for each scanner type as reported by the WMH challenge website.^3^ Furthermore, the website shows boxplots for all 5 metrics comparing the results obtained for each scanner.

**Table 5:** WMH challenge results. The average performance of SegAE on each of the metrics of the WMH challenge on test data from each scanner, and the weighted average of the scores achieved on images from each scanner type for each metric.2.f.

	DSC	H95	AVD	L-TPR	L-F1
Utrecht (n=30)	0.57	31.57	79.90	0.35	0.30
Singapore (n=30)	0.67	17.70	16.61	0.25	0.32
AMS GE3T (n=30)	0.65	16.56	22.41	0.39	0.48
AMS GE1.5T (n=10)	0.64	17.04	17.76	0.31	0.44
AMS PETMR (n=10)	0.53	54.87	111.59	0.40	0.23
Weighted average	0.62	24.49	44.19	0.33	0.36

## 5 Discussion

**Fig. 10:**
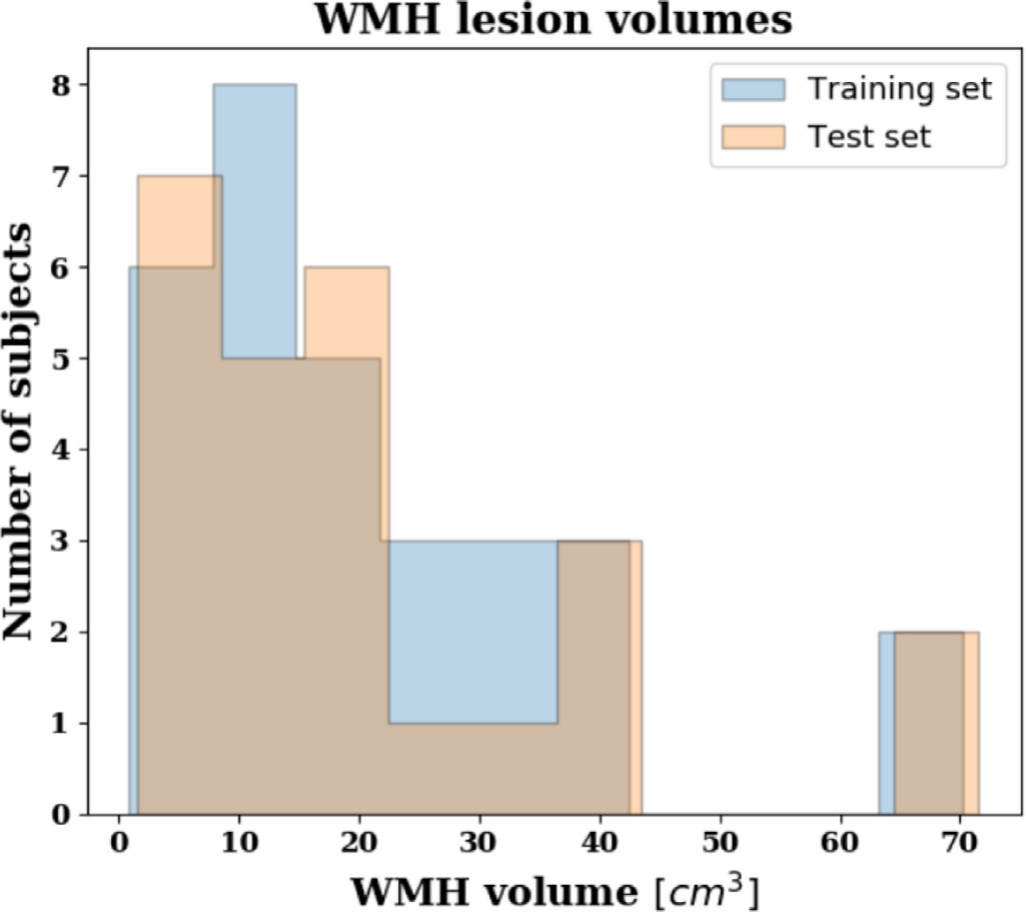
A histogram showing the WMH lesion volumes of the AGES-Reykjavik training (blue) and test (peach) sets. The volumes were predicted from SegAE since manual delineations do not exist for the training images.

Given a training set of brain MRIs, SegAE learns the segmentation of the predominant materials that make up these images. Whether a material is predominant depends on the contrast and abundance of the material in the image. In our case, it was sufficient to randomly sample brain MRIs from the population of elderly subjects to get WMHs as one of those materials (see the lesion load of our training and test data in Fig. 10). After training, the segmentations of WMHs, GM, WM and CSF generated by SegAE were visually validated, and if the training was successful, SegAE could be used to directly generate segmentations for new images that were not in the training set. We trained and evaluated SegAE on brain images from a population study with a highly variable WMH lesion load, from almost no WMHs to a very high WMH lesion load. The segmentation results indicate the robustness of our method regardless of lesion load and location.

An advantage of SegAE is that we do not need a large data set of training subjects because our unsupervised methodology is based on the intensity features that are shared between all the sequences used as training images. Then after training the method on images from 30 subjects from the AGES-Reykjavik data set, it can be used to segment the remaining subjects (4781 subjects) extremely fast. The average run time per scan in the AGES-Reykjavik test set was 19 seconds using a GTX1080 Ti GPU.

The DSC, AVD, H95, L-TPR, and L-F1 were used as evaluation metrics in the WMH challenge, and we used the same metrics to evaluate our results on the AGES-Reykjavik data set for consistency. On the AGES-Reykjavik test set, we compared the method with three alternative WMH segmentation methods, i.e., LPA, LGA, and ANNC. SegAE achieved the best average DSC, L-TPR, and L-F1 scores, while LPA achieved the best average H95 score (cf. Table 4). WMHs are not an intact structure so the H95 score is not very informative, however, a high H95 might suggest skullstripping errors causing oversegmentation of WMHs at the brain boundary. LGA achieved the best average AVD score despite having a volume correlation of only 0.78, seemingly because the AVD score penalizes undersegmentations less than oversegmentations, as mentioned in Kuijf et al. (2019). SegAE and ANNC achieved the highest volume correlation (r=0.98), however, ANNC seems to systematically overestimate the lesion volumes, as indicated in Fig. 8, hence SegAE achieves a significantly better AVD (p  <  0.01) than ANNC. A systematic overestimation of WMHs can explain the higher AVD and high correlation in ANNC because the correlation coefficient is bias and scale invariant.

The DSC is more sensitive to errors in segmentation of small structures, so DSC was plotted with manual volumes as a reference in Fig. 7. Bottom part of Fig. 7 demonstrates the robustness of SegAE to a variety of WMH volumes and in Fig. 6, bottom row, we visually verify that the segmentation where SegAE achieves the lowest DSC is not a failure.

The results on the MICCAI 2017 WMH segmentation challenge test set can be seen in Table 5. On the challenge website^4^, methods are ranked according to the average rank for all metrics, but methods can also be compared for each metric individually. SegAE is currently the best performing unsupervised method, using either the website’s ranking system or the average DSC. The method also compares favorably to some supervised methods.

Assuming that the true WMH segmentations from the WMH challenge and the AGES-Reykjavik data set come from the same distribution, then comparing average scores in Tables 4 and 5 shows that SegAE performs better on the AGES-Reykjavik test set than the WMH challenge test set. This is not surprising, since the FLAIR images in the AGES-Reykjavik data set have better contrast between WMH and GM, and T2-w and PD-w images are used in addition to the FLAIR and T1-w images for training the AGES-Reykjavik network. Fig. 5 in Supplementary materials shows that using only FLAIR images or T1-w and FLAIR images for training the AGES-Reykjavik data set can increase susceptibility to artifacts. Visual inspection of the WMH challenge training images shows that some small, low intensity WMHs are not detected (see Fig. 9, middle and bottom rows). This could explain the substantially lower L-TPR and L-F1 scores for the WMH challenge test set than the AGES-Reykjavik test set. Furthermore, during training of SegAE on the WMH challenge training set, data from three different scanners are used, while the method is tested on data from five different scanners. This could interfere with training if the image contrast in the different scanners differs, since SegAE reconstructs all training images by the same weighted sum of the segmentation of materials present in the images during training. We note that the meninges class did not appear in the WMH challenge model, possibly due to the absence of T2-w or PD-w images. Finally, it is unknown whether any WMH segmentation errors in the WMH challenge test set are caused by errors in skullstripping, since the test set and its results are blinded. The much higher H95 and AVD for some images from Utrecht and the AMS PETMR results may suggest that this might be the case.

Although segmentation of WMHs of presumed vascular origin is the main focus of this paper, hyperintense lesions in FLAIR images can have other causes, such as multiple sclerosis (MS) and traumatic brain injury (TBI). Methods for unsupervised segmentation of FLAIR hyperintensities are often used interchangeably (Bowles et al., 2017) and we believe that the proposed method should be able to segment any lesions with similar intensities in the MRI sequences that we use.

## 6 Conclusions

We have presented SegAE, a CNN architecture that can be trained in an unsupervised manner to segment WMHs in brain MRIs. We evaluated the WMH segmentation from the proposed method on two separate data sets acquired from six different scanners, i.e. the AGES-Reykjavik data set and the MICCAI 2017 WMH segmentation challenge data set, using ground truth manual WMH labels. For the AGES-Reykjavik test set the method was compared with three alternative WMH segmentation methods, i.e., LPA, LGA, and ANNC. SegAE achieved the best average DSC, L-TPR, and L-F1 scores, while LPA achieved the best H95 score, and LGA the best AVD score. SegAE achieved a WMH lesion volume correlation of 0.98. The results on the MICCAI 2017 WMH segmentation challenge test set can be seen in Table 5. The scores can be compared with any method sent to the WMH segmentation challenge via the WMH challenge website^5^.

## Appendix A Supplementary materials

Supplementary Data S1 Supplementary Raw Research Data. This is open data under the CC BY license http://creativecommons.org/licenses/by/4.0/ Supplementary Data S1

## Footnotes

1 https://wmh.isi.uu.nl/

2 www.statisticalmodelling.de/lst.html

3 https://wmh.isi.uu.nl/results/himinn/

4 https://wmh.isi.uu.nl/

5 https://wmh.isi.uu.nl/
