# Supplementary material for: SegAE: Unsupervised white matter lesion segmentation from brain MRIs using a CNN autoencoder
Source: Neuroimage Clin. 2019 Nov 9;24:102085. doi: 10.1016/j.nicl.2019.102085 (PMC6861597; doi:10.1016/j.nicl.2019.102085)
Supplement: Supplementary Data S1 — Supplementary Raw Research Data. This is open data under the CC BY license http://creativecommons.org/licenses/by/4.0/ [file mmc1.pdf]

# SegAE: Unsupervised white matter lesion segmentation from brain MRIs using a CNN autoencoder: Supplementary materials

Hans E. Atlason*
Dept. of Electrical and Computer Engineering, University of Iceland

November 11, 2019

## 1 SegAE comparison

### 1.1 Old vs. new

A preliminary version of SegAE was presented earlier in conference format [1]. Although the CNN architecture remains the same, the input MRIs and training methodology was substantially modified to improve the performance of the method presented here. The major modifications and the motivation behind each one of them are listed below:

1. *A scale-invariant loss function and a new regularizer to stabilize training*: The loss function used in the preliminary version presented in our conference paper was $L = (Y^3 - \hat{Y}^3)$, where $Y$ is a 3D patch from a FLAIR image and $\hat{Y}^3$ is the corresponding estimated FLAIR image patch. The power of 3 was used to put more weight on the FLAIR hyperintensities, however, there are several drawbacks with this approach: a) It would not work as intended when we add new MRI sequences because white matter (WM) and cerebrospinal fluid (CSF) have high intensity in T1-w and T2-w images, respectively; b) this image transformation tends to amplify noise and skew the relative tissue intensities; c) difference between true vs. predicted patches depends on the intensity scale, which makes training noisy because of imperfect image normalization. This effect was to some extent mitigated by using higher beta parameters in the Adam optimizer to reduce learning noise. In the new version of SegAE presented here we use the Cosine proximity function to construct a cost function, which is scale-invariant and does not cause the aforementioned problems (see details in the main text).

*See the main text for the complete author list

The preliminary version presented in the conference paper had no regularizer as part of the cost function; only scaling of the CNN activations before applying the Softmax function. The reasoning for this was that the Softmax function approaches the argmax function when the input is in the range $[0, \inf)$. However, the CNN eventually learns weights that give non-binary Softmax outputs to lower the cost, so early stopping was needed. In the method presented here, we have an explicit regularization term in the cost function so the solution converges to the expected segmentations.

2. *More MR sequences contributing to the calculation of the loss function*: In the preliminary version we used only FLAIR images for the calculation of the loss function. In the new version presented in this paper we use T1-w, T2-w, and FLAIR, which makes the method more robust to inhomogeneity artifacts and noise. A comparison of the method using fewer MR sequences is shown below in Section 1.4.

3. *An inhomogeneity correction performed during the training phase*: In the previous paper we didn't use any inhomogeneity correction because we found that the N4 bias-correction [8] tended to degrade the WMH lesion segmentations. This resulted in over-segmentation of WMHs in areas around the brain cortex that were removed afterwards with a morphologically eroded brainmask. This is explained in the conference paper and is an obvious disadvantage of the previous method in terms of complexity, processing time, and sensitivity to WMHs (see Figure 1 (b)). In the SegAE version presented here we perform inhomogeneity correction during the training phase, as described below.

## 1.2 Improved inhomogeneity correction

This journal article discusses the N4 bias-correction method and how it can be used to process FLAIR images with WMHs. For the AGES-Reykjavik data set we observed that when using the default settings of N4 (single mesh over the entire domain) the inhomogeneity artifacts were not adequately removed and decreasing the B-spline distance parameter tended to degrade the lesions (see Figure 2). This effect is demonstrated in Figure 2 (c) and (d), where we decreased the B-spline distance to 150 mm and 100 mm, respectively, leading to substantial improvement of the inhomogeneity artifacts in the cortical regions, however, at the same time causing the WM lesions to lose contrast. To address this we used the pure-tissue probability mask option proposed in [9]. This enabled us to use N4 for estimating the bias field without affecting the WMHs, by excluding the WMH regions when doing the bias field estimation.

Furthermore, the T1-w and T2-w images of the AGES-Reykjavik data set were processed differently in the proposed method: They were intensity transformed using the corresponding PD-w images to correct the bias-field, in particular, inhomogeneity artifacts in the lateral ventricles in the T2-w images (see Figure 3 in the main text), and for contrast enhancement of the T1-w images.

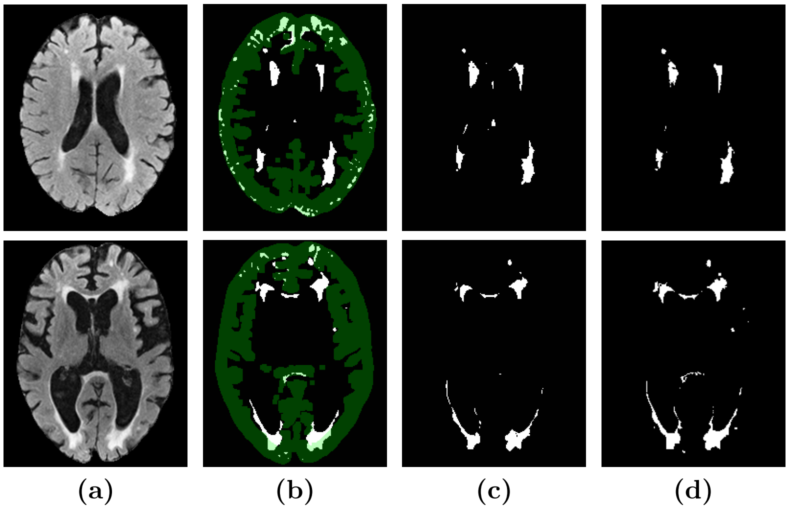

Figure 1: The figure shows **(a)** a FLAIR image, **(b)** the output of the preliminary version of SegAE [1]; green overlay shows the areas that were removed in a special post-processing step using a morphologically eroded brainmask (without sulcal CSF) from FreeSurfer, **(c)** the result from the proposed way of training SegAE, and **(d)** the manually delineated WMHs.

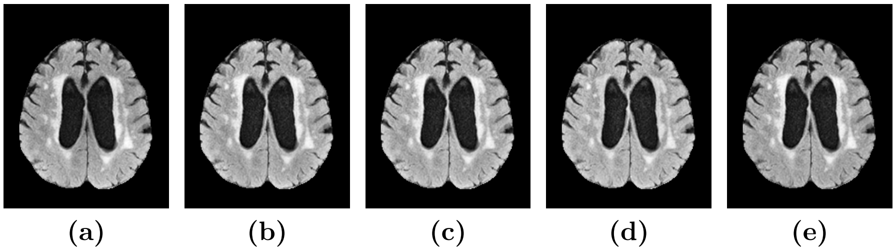

Figure 2: Comparison of the N4 bias-correction method when using different B-spline distance showing **(a)** the original FLAIR image (after skullstripping), **(b)** showing the default settings of N4 (single mesh), **(c)** the FLAIR image when using 150 mm distance, **(d)** the FLAIR image when using 100 mm distance, and **(e)** the FLAIR image when using a pure-tissue probability mask.

Comparison of three SegAE models, trained to reconstruct images that have been bias corrected with 1) standard N4, 2) N4 with pure-tissue probability mask, and 3) N4 with pure-tissue probability mask for processing the FLAIR image but intensity transformation with a PD-w image for the T1-w and T2-w images, can be seen in Figure 3.

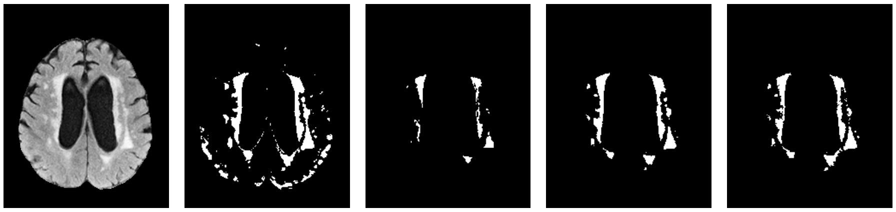

**(a)** **(b)** **(c)** **(d)** **(e)**

Figure 3: The figure shows the WMH output of SegAE trained on **(b)** T1-w, T2-w, and FLAIR images after standard N4 bias-correction; (c) T1-w, T2-w, and FLAIR after N4 correction with pure-tissue probability masks; and (d) intensity transformed T1-w and T2-w images (using corresponding PD-w images) and N4 bias-corrected FLAIR images using pure-tissue probability masks. Figure (a) shows the FLAIR image and (e) shows the manually delineated WMH mask for comparison.

## 1.3 DSC and volume comparison

A comparison of the lesion volumes and Dice Similarity Coefficient (DSC) for the old SegAE scheme, SegAE using standard N4 bias-correction, and the proposed SegAE for 15 subjects (same subjects as in [1]) can be seen in Figure 4. Using standard N4 bias correction gives lower DSC in all cases, and using the old method of training and post-processing SegAE gives lower DSC in 10 out of 15 cases.

## 1.4 Number of input sequences

In this article we present results on two separate data sets, i.e. the AGES-Reykjavik data set and the WMH challenge data set. The AGES-Reykjavik data set consists of T1-w, T2-w, FLAIR, and PD-w images, while the WMH challenge data set comprises only T1-w and FLAIR images.

To evaluate the effects of using different number of input sequences we conducted several experiments by training SegAE using 1) only FLAIR images, 2) T1-w and FLAIR images and, 3) T1-w, T2-w, and FLAIR images (and PD-w images for image enhancement). A visual comparison of the WMH segmentations can be seen in Figure 5. Using more input sequences, if available, seems to improve the robustness to cases with severe inhomogeneity artifacts that can not be completely removed with the N4 bias-correction, and improve robustness to noise.

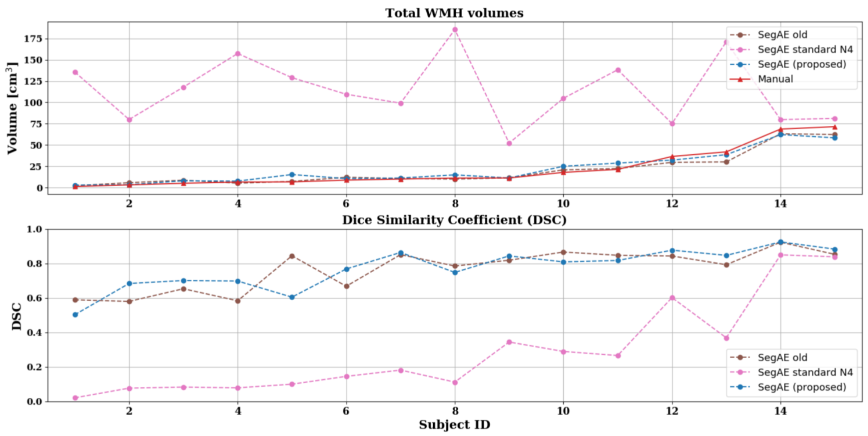


Figure 4: The top graph shows the overall WMH volume for the manual masks (red) and masks generated by old SegAE (brown, dotted), SegAE with standard N4 (pink, dotted), and proposed SegAE (blue, dotted), ordered by the volume of the manual masks. The bottom graph shows the DSC for the same methods compared with the manual masks.

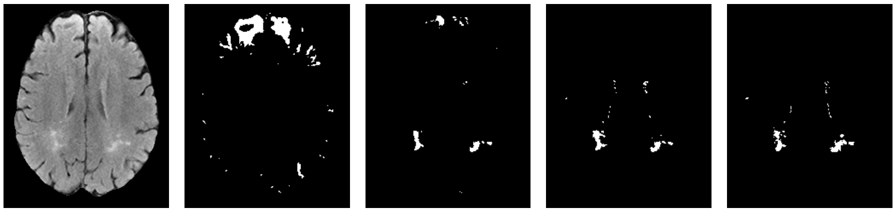

**(a)** **(b)** **(c)** **(d)** **(e)**

Figure 5: WMH segmentation output from SegAE using different input sequences. **(a)** the FLAIR image; **(b)** using only FLAIR images as input; **(c)** using T1-w, and FLAIR images as input; **(d)** the proposed SegAE using T1-w, T2-w, and FLAIR images as input; and **(e)** the manually delineated mask.

# 2 WMH challenge models

SegAE was submitted to the WMH challenge (MICCAI 2017 [5]). Training data from three scanners were provided; GE3T (20 subjects), Singapore (20 subjects), and Utrecht (20 subjects). We trained SegAE on training data from all three scanners simultaneously and submitted this model to the challenge.

During training, SegAE is sensitive to the contrast of the training images, so here we explore whether the performance on the training set would improve if SegAE was trained on data from each scanner separately. Figure 6 shows boxplots of the DSC scores achieved for each training set separately when SegAE models are trained on each training set separately, as well as all the data simultaneously, as was done in the challenge. These results suggest that training

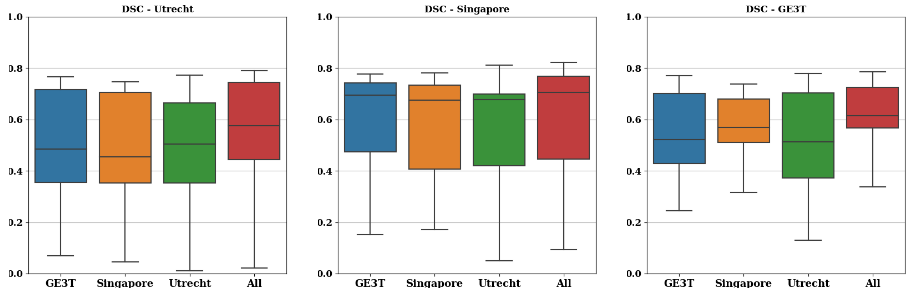


**(a)** **(b)** **(c)**

Figure 6: DSC comparision of SegAE models trained using only images from GE3T (blue), Singapore (orange), Utrecht (green), as well as images from all scanners combined (red). **(a)** shows the results evaluated on images from the Utrecht training set, **(b)** shows results evaluated on the Singapore training set, and **(c)** shows results evaluated on the GE3T training set.

SegAE using all available training data improves the performance on all the WMH challenge data sets, even though the data come from different scanners.

# 3 Skullstripping U-net

## 3.1 CNN architecture

For skull removal of the unseen WMH challenge test data (performed by the WMH challenge team), we deveoloped a special skullstripping U-net that we submitted with our segmentation method, SegAE, to the challenge. The skullstripping network is a three dimensional (3D) convolutional neural network with a U-net [6] like architecture. The input consists of 3D patches of size $80 \times 80 \times 40$ from FLAIR and T1-weighted images and the output is a segmentation of the brain and background.

The CNN consists of 3D convolutional layers (kernel size $3 \times 3 \times 3$) followed by leaky rectified linear units (LReLU) activation functions and batch normalization layers. Downsampling is performed with $2 \times 2 \times 2$ strided convolutions, but $2 \times 2 \times 2$ upsampling is performed to obtain an output of the same size as the input. The network architecture can be seen in Figure 7.

## 3.2 Training and prediction

The network was trained using the training images of the WMH segmentation challenge [5] and the corresponding brainmask generated by MONSTR [7]. Input images were intensity normalized by dividing by the 99th percentile of the non-zero elements of the image. The training images were cropped to the smallest cuboid containing the brain and patches from the images were acquired with a stride of 40 voxels. Only 50% of the extracted patches, which had the fewest

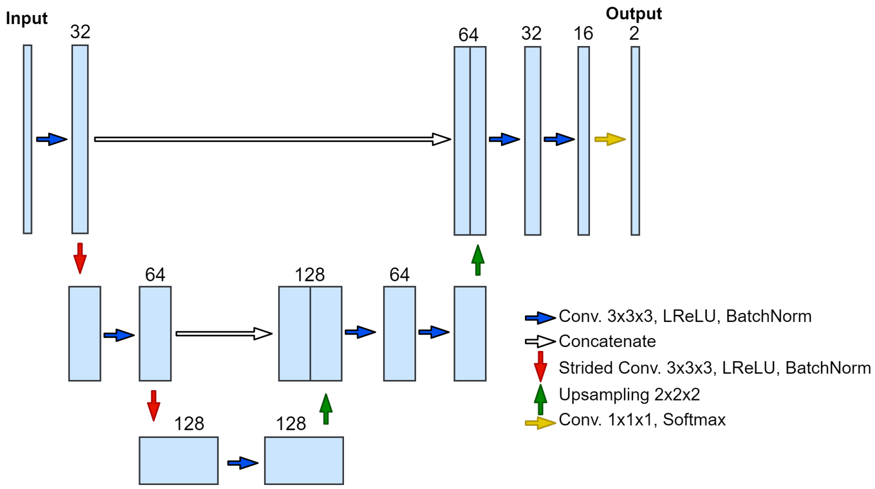


Figure 7: The skullstripping U-net. The input comprises large 3D patches from FLAIR and T1-weigted MRIs.

background voxels, were used for training. A weighted categorical crossentropy function was used for training the network. A GTX1080 Ti GPU was used to train the network for 160 epochs with a learning rate of 0.0001 using the Adam optimizer [4], with Nesterov momentum [2], with $\beta_1 = 0.9$, $\beta_2 = 0.999$, schedule decay of 0.004, and a batch size of one. During training, Gaussian noise with a standard deviation of 0.05 and zero mean was added to the input patches, and different scalar values drawn from a Gaussian distribution with a mean value of 1 and standard deviation of 0.5 were multiplied with each channel of the input patches to improve the invariance of the network to possibly inconsistent normalization of unseen images. All weights of the convolutional network were initialized using Glorot uniform initialization [3] and biases were initialized as zero. LReLU activation functions had a slope of 0.1 for the negative part.

After training, the brainmask output of the CNN was assembled to the size of the original volume using the average of the overlapping patches, and a threshold of 0.5 was used to binarize the brainmask.

# References


[1] Atlason, H.E., Love, A., Sigurdsson, S., Gudnason, V., Ellingsen, L.M.: Unsupervised brain lesion segmentation from MRI using a convolutional autoencoder. In: Medical Imaging 2019: Image Processing. vol. 10949, p. 109491H. International Society for Optics and Photonics (2019)

[2] Dozat, T.: Incorporating nesterov momentum into adam (2016)

[3] Glorot, X., Bengio, Y.: Understanding the difficulty of training deep feedforward neural networks. In: Proceedings of the thirteenth international conference on artificial intelligence and statistics. pp. 249–256 (2010)

[4] Kingma, D.P., Ba, J.: Adam: A method for stochastic optimization. arXiv preprint arXiv:1412.6980 (2014)

[5] Kuijf, H.J., Biesbroek, J.M., de Bresser, J., Heinen, R., Andermatt, S., Bento, M., Berseth, M., Belyaev, M., Cardoso, M.J., Casamitjana, A., et al.: Standardized assessment of automatic segmentation of white matter hyperintensities; results of the wmh segmentation challenge. IEEE transactions on medical imaging (2019)

[6] Ronneberger, O., Fischer, P., Brox, T.: U-net: Convolutional networks for biomedical image segmentation. In: International Conference on Medical image computing and computer-assisted intervention. pp. 234–241. Springer (2015)

[7] Roy, S., Butman, J.A., Pham, D.L.: Robust skull stripping using multiple MR image contrasts insensitive to pathology. Neuroimage **146**, 132–147 (Feb 2017)

[8] Tustison, N.J., Avants, B.B., Cook, P.A., Zheng, Y., Egan, A., Yushkevich, P.A., Gee, J.C.: N4ITK: improved N3 bias correction. IEEE transactions on medical imaging **29**(6), 1310–1320 (2010)

[9] Tustison, N.J., Cook, P.A., Klein, A., Song, G., Das, S.R., Duda, J.T., Kandel, B.M., van Strien, N., Stone, J.R., Gee, J.C., et al.: Large-scale evaluation of ants and freesurfer cortical thickness measurements. Neuroimage **99**, 166–179 (2014)
